# Associations of Enteric Protein Loss, Vaccine Response, Micronutrient Deficiency, and Maternal Depressive Symptoms with Deviance in Childhood Linear Growth: Results from a Multicountry Birth Cohort Study

**DOI:** 10.4269/ajtmh.21-0403

**Published:** 2022-04-11

**Authors:** Subhasish Das, Visnu Pritom Chowdhury, Md. Amran Gazi, Shah Mohammad Fahim, Md. Ashraful Alam, Mustafa Mahfuz, Esto Mduma, Tahmeed Ahmed

**Affiliations:** ^1^ Nutrition and Clinical Services Division, International Centre for Diarrhoeal Disease Research, Bangladesh (icddr,b), Dhaka, Bangladesh;; ^2^ Liggins Institute, University of Auckland, Auckland, New Zealand;; ^3^ Haydom Global Health Institute, Haydom, Tanzania;; ^4^ James P. Grant School of Public Health, BRAC University, Dhaka, Bangladesh;; ^5^ Department of Global Health, University of Washington, Seattle, Washington;; ^6^ Department of Public Health Nutrition, James P. Grant School of Public Health, BRAC University, Dhaka, Bangladesh;; ^7^ Office of Executive Director, icddr,b, Dhaka, Bangladesh

## Abstract

We identified the determinants of positive (children who had a birth weight < 2.5 kg and/or maternal height < 145 cm but were nonstunted at 24 months of age) and negative (children who had a birth weight ≥ 2.5 kg and maternal height ≥ 145 cm but were stunted at 24 months of age) deviance in childhood linear growth. We found that socioeconomic status (β = 1.54, *P* < 0.01), serum retinol (β = 0.05, *P* < 0.01), hemoglobin (β = 0.36, *P* < 0.01), length-for-age Z-score (LAZ) at birth (β = 0.47, *P* < 0.01), and tetanus vaccine titer (β = 0.182, *P* < 0.05) were positively and maternal depressive symptom (β = –0.05, *P* < 0.01), serum ferritin (β = –0.03, *P* < 0.01), male sex (β = –1.08, *P* < 0.01), and α1-antitrypsin (β = –0.81, *P* < 0.01) were negatively associated with positive deviance. Further, diarrhea episodes (β = 0.02, *P* < 0.01), male sex (β = 0.72, *P* < 0.01), and α1-antitrypsin (β = 0.67, *P* < 0.01) were positively and hemoglobin (β= –0.28, *P* < 0.01), soluble transferrin receptor level (β = –0.15, *P* < 0.01), and LAZ score at birth (β = –0.90, *P* < 0.01) were negatively associated with negative deviance. To summarize, enteric protein loss, micronutrient deficiency, vaccine responses and maternal depressive symptoms were associated with linear growth deviance in early childhood. In such a background, public health approaches aimed at reducing the risk of intestinal inflammation and altered gut permeability could prove fruitful in ensuring desired linear growth in children. In addition, maternal mental health issue should also be considered, especially for promoting better nutritional status in children in the context of linear growth deviance.

## INTRODUCTION

Linear growth faltering or stunting (length-for-age Z-score < –2) is an indicator of chronic undernutrition in children.^1^ It is one of the most prevalent forms of childhood malnutrition that affects 155 million children under age 5 years globally. Shorter maternal height and low birth weight are the two most prominent risk factors of linear growth faltering in children.^2^^–^^5^ The risk is even higher when low‐birth-weight children are born to short‐stature mothers.^6^ However, several studies have reported that despite the presence of the factors (shorter maternal height and low birth weight), there were children who successfully avert linear growth faltering.^7^^,^^8^ Similarly, there were children who failed to attain optimal linear growth even in the absence of the aforementioned factors. Children demonstrating such deviances in growth and nutritional status could be regarded as positive and negative deviants that result from social, behavioral, and physiological adaptability to nutritional stress.^9^^–^^11^

To elaborate, the term *positive deviance* explains the adaptive responses for satisfactory child growth under growth demotivating environments, such as maternal undernutrition, poor complementary feeding, poor childcare, lack of maternal education, lower number of visits to doctor during pregnancy, low socioeconomic status, among other factors.^11^^,^^12^ *Negative deviance* is described as the failure of children to grow satisfactorily, even under favorable conditions such as maternal wealth and male sex, for eample.^9^^,^^10^

Positive deviance approaches have been used in low- and middle-income country settings in programmatic contexts to identify behaviors and practices associated to it with the aim of guiding program development.^13^^,^^14^ However, none of the efforts counted the roles of gut enteropathy, enteric protein loss, vaccine responses, or other biological factors to predict linear growth deviance in children. Moreover, factors leading to positive deviance might not always be the reverse of the factors of negative deviance.^10^ If the programs that were delivered in the context of growth deviance were developed considering the aforementioned biological features, it could add more value in alleviating malnutrition. Hence, we did a deviance inquiry using the data collected from the Malnutrition and Enteric Diseases (MAL-ED) multicountry birth cohort study, intending to identify the social, behavioral, physiological, and biological factors responsible for positive and negative deviance in childhood linear growth.

## MATERIALS AND METHODS

### Study setting and data source.

We gathered data from a multicountry birth cohort study named “Etiology, Risk Factors, and Interactions of Enteric Infections and Malnutrition and the Consequences for Child Health” (MAL-ED) study. This study was conducted at eight sites across three continents. In this analysis, we included data from six MAL-ED sites: Dhaka, Bangladesh, and Vellore, India in Asia; Fortaleza, Brazil, and Loreto, Peru, in the Americas; and Venda, South Africa, and Haydom, Tanzania, in Africa. Children were enrolled from November 2, 2009 to February 28, 2014, within 7 days of their birth and were followed uniformly up to 24 months of age after predefined validated protocols.^15^^–^^17^ The MAL-ED study protocol received ethical approvals from the institutional review boards of the respective sites. Informed written consent was taken from the parents or legal guardians of the enrolled children after informing them about the study objective and related methods.

### Outcome variable: operational definition of deviance groups.

An analysis of the MAL-ED birth cohort study reported two nonmodifiable characteristics—maternal height and birth weight—as contributing significantly in modifying linear growth in children throughout the first 24 months after birth.^2^ Hence, based on the stunting status at 24 months and the maternal height and birth weight of the children, we divided 1,092 children of MAL-ED birth cohort to four deviant groups (Supplemental File 1; Tables 1 and 2):

**Table 1:** Sociodemographic characteristics of the participants

	NPD (*N* = 117)	PD (*N* = 79)	All (PD+NPD; *N* = 196)	NND (*N* = 548)	ND (*N* = 349)	All (NND+ND; *N* = 897)
	Mean (SD)					
Birthweight (in kg)	2.6 (0.46)	2.7 (0.50)	2.6 (0.48)	3.3 (0.48)	3.2 (0.41)	3.3 (0.46)
Mother’s height (in cm)	150 (5.8)	150 (5.7)	150 (5.8)	160 (5.9)	150 (5.7)	150 (5.9)
LAZ-score at birth	−1.9 (1.1)	−1.5 (1.1)	−1.7 (1.1)	−0.54 (0.94)	−1.1 (0.89)	−0.76 (0.96)
LAZ-score at 24 months	−3.0 (0.72)	−1.2 (0.71)	−2.3 (1.1)	−0.94 (0.89)	−2.8 (0.64)	−1.7 (1.2)
EBF days	78 (53)	77 (61)	77 (56)	57 (50)	55 (47)	56 (49)
WAMI index*	0.45 (0.17)	0.56 (0.18)	0.50 (0.18)	0.63 (0.21)	0.45 (0.24)	0.56 (0.23)
SRQ**-**20 score	5.1 (3.9)	4.5 (3.3)	4.8 (3.6)	3.6 (2.9)	3.7 (3.1)	3.7 (2.9)

EBF = exclusive breastfeeding; LAZ-score = length-for-age Z-score; ND = negative deviant; NND = nonnegative deviant; NPD = nonpositive deviant; PD = positive deviant; SRQ-20: Self-Reporting Questionnaire–20.

* WAMI index: WAMI index (Water, sanitation, hygiene, Asset, Maternal education, and Income index, ranging from 0 to 1) is a socioeconomic status index that includes access to improved water and sanitation, eight selected assets, maternal education, and household income was used as a representative of socio-economic status of the households.

**Table 2:** Factors associated with the chances of being positive and negative deviants

Outcome variable: Positive deviant, yes (*N* = 79) vs. no (*N* = 117))				
Variables	Unadjusted		Adjusted	
	Coefficient (95% CI*)	*P* value	Coefficient (95% CI)	*P* value
Energy from protein (Kcal/day)	0.03 (0.02 to 0.03)	< 0.001	0.02 (−0.01 to 0.04)	0.186
WAMI*	2.49 (1.90 to 3.08)	< 0.001	1.54 (0.80 to 2.28)	< 0.001
SRQ-20***	−0.04 (−0.07 to −0.02)	< 0.001	−0.05 (−0.07 to −0.02)	< 0.001
L-M Ratio****	−0.48 (−0.76 to −0.19)	0.001	−0.14 (−0.49 to 0.21)	0.422
Hemoglobin (g/dL)	0.28 (0.21 to 0.35)	< 0.001	0.36 (0.26 to 0.45)	< 0.001
Retinol (µmol/L)	0.07 (0.06 to 0.08)	< 0.001	0.05 (0.04 to 0.07)	< 0.001
Ferritin, ng/mL	−0.01 (–0.01 to 0.00)	0.001	−0.03 (−0.03 to −0.02)	< 0.001
Sex	−0.90 (–1.05 to −0.75)	< 0.001	−1.08 (−1.26 to −0.90)	< 0.001
Myeloperoxidase, ng/ml	0.000012 (0.000022 to 0.0000017)	0.022	0.00003 (0.00005 to 0.00002)	< 0.001
Neopterin, nmol/L	0.00016 (0.00004 to 0.00024)	< 0.001	0.00003 (0.00012 to −0.00007)	0.600
α-1-antitrypsin, mg/g	−0.53 (−0.86 to −0.19)	0.002	−0.81 (−1.22 to −0.41)	< 0.001
LAZ score***** at birth	0.33 (0.26 to 0.40)	< 0.001	0.47 (0.39 to 0.56)	< 0.001

Outcome variable: Negative deviant, yes (*N* = 349) vs. no (*N* = 548)				
	Unadjusted		Adjusted	
Variables	Coefficient (95% CI)	*P* value	Coefficient (95% CI)	*P* value
Energy from protein	−0.02 (–0.03 to −0.01)	< 0.001	−0.02 (–0.03 to 0.00)	0.006
Diarrhea episodes	0.01 (0.00 to 0.02)	0.011	0.02 (0.01 to 0.03)	< 0.001
EBF days	0.00 (0.00 to 0.00)	< 0.001	0.00 (0.00 to 0.00)	< 0.001
Hemoglobin (g/dL)	−0.21 (–0.25 to −0.17)	< 0.001	−0.28 (–0.32 to −0.23)	< 0.001
Retinol (µmol/L)	−0.02 (–0.02 to −0.01)	< 0.001	0.00 (–0.01 to 0.00)	0.193
Transferrin receptor, ng/mL	−0.06 (–0.07 to −0.04)	< 0.001	−0.15 (–0.17 to −0.13)	< 0.001
Ferritin, µg/L	0.01 (0.00 to 0.01)	< 0.001	0.01 (0.01 to 0.01)	< 0.001
Sex	0.65 (0.58 to 0.73)	< 0.001	0.72 (0.63 to 0.80)	< 0.001
Myeloperoxidase, ng/mL	0.00002 (0.00003 to 0.00001)	< 0.001	0.00002 (0.00002 to 0.00001)	< 0.001
Neopterin, nmol/L	−0.00004 (–0.00001 to −0.00006)	0.008	−0.00006 (–0.00003 to −0.00009)	< 0.001
α-1-antitrypsin, mg/g	0.50 (0.33 to 0.67)	< 0.001	0.67 (0.48 to 0.86)	< 0.001
LAZ score at birth	−0.81 (–0.85 to −0.76)	< 0.001	−0.90 (–0.95 to −0.85)	< 0.001

CI = confidence interval; EBF = exclusive breastfeeding; LAZ-score = length-for-age Z-score; L-M ratio = lactulose-mannitol ratio; SRQ-20 score = Self Reporting Questionnaire–20.

* WAMI index: WAMI index (Water, sanitation, hygiene, Asset, Maternal education, and Income index, ranging from 0 to 1) is a socioeconomic status index that includes access to improved water and sanitation, eight selected assets, maternal education, and household income was used as a representative of socioeconomic status of the households.

- Positive deviant (PD): Seventy-nine children who had a birth weight < 2.5 kg and/or maternal height < 145 cm but were nonstunted at 24 months of age
- Nonpositive deviant (NPD): 116 children who had a birth weight < 2.5 kg and/or maternal height < 145 cm and were stunted at 24 months of age
- Negative deviant (ND): A total of 349 children who had a birth weight ≥ 2.5 kg and maternal height ≥ 145 cm but were stunted at 24 months of age
- Non-negative deviant (NND): A total of 548 children who had a birth weight ≥ 2.5 kg and maternal height ≥ 145 cm and were non-stunted at 24 months of age.

Here, the length-for-age Z-score (LAZ score) for each child was determined using the WHO 2006 Child Growth Standards and stunting was defined as LAZ score < –2 SD of the WHO Child Growth Standards median.^18^ Enrollment weight, taken within the first 7 days of birth, was used as the surrogate of birth weight.

### Predictor variables.

Data on predictor variables were gathered from eight domains: demographic and socioeconomic indicators, dietary intake, maternal depressive symptoms, morbidity, gut inflammation, gut integrity, serum micronutrient status, and vaccine response status.

#### Demographic and socioeconomic indicators.

Socioeconomic data was collected at 6, 12, 18, and 24 months of age of a child. The WAMI index (Water, sanitation, hygiene, Asset, Maternal education, and Income index, ranging from 0 to 1), a socioeconomic status index that includes access to improved water and sanitation, eight selected assets (separate room for a kitchen, household bank account, mattress, TV, refrigerator, people per room, table, chair or bench), maternal education, and household income was used as a representative of socioeconomic status of the households.^19^ A higher WAMI index means a better socioeconomic status. The statistical analysis to calculate WAMI score was done in two phases. First, the best approach for selecting and weighting household assets as a proxy for wealth was identified. Four approaches for measuring wealth (maternal education, principal components analysis, multidimensional poverty index, and a novel variable selection approach based on the use of random forest algorithm) were compared. Second, the selected wealth measure was combined with other relevant variables to form the index.^19^

#### Assessment of dietary intake.

For assessing dietary intake of the children, we collected 24-hour dietary recall data monthly from the ninth month onward using a 24-hour multiple-pass dietary recall approach.^20^ The interviews were conducted on nonconsecutive days, and one out of every four recalls was conducted on a weekend. The total amount of energy taken from protein intake was measured from the dietary intake data using a locally adapted food composition table. Data collectors asked the mother about the liquids the child consumed during the past 24 hours and whether the response followed the WHO definition of exclusive breastfeeding (EBF; no other foods or drink, not even water, except breast milk, including expressed milk, oral rehydration solution, vitamins, minerals, and medicine syrups); if so, the child was considered as exclusively breastfed. Instead of EBF status (yes versus no), EBF days were used during data analysis because it specifies the extent of EBF to specific number of days.

#### Maternal depressive symptoms.

Trained fieldworkers recorded the depressive symptoms of a mother using Self Reporting Questionnaire-20 (SRQ-20).^21^ The questionnaire, comprising 20 binary (yes versus no) type of questions, is developed by the WHO for use in developing countries and is designed to assess maternal psychological adjustment related to depressive symptoms.^22^^,^^23^

#### Morbidity, gut inflammation, and gut integrity.

Data on diarrheal episodes were collected twice a week. Diarrhea is defined as having three or more loose stools in 24 hours or at least one loose stool with blood reported by the mother, and a diarrheal episode is defined as being separated from another episode by at least two or more diarrhea-free days.^24^ The markers of gut inflammation (Alpha-1-antitrypsin; [A1AT; ELISA kit: Bio vendor, Chandler, NC], neopterin [Neo; ELISA kit: GenWay Biotech, San Diego, CA], and myeloperoxidase [MPO; ELISA kit: Alpco, Salem, NH]; measured in stool at 7, 15, and 24 months) and gut integrity (lactulose-mannitol ratio, LM ratio; measured in urine at 3, 6, 9, and 15 months) were measured from all the children following a standard protocol.^15^ All the biomarkers were measured longitudinally, and the mean of all values were used for data analyses.

#### Micronutrient status and vaccine responses.

Blood samples for measuring serum zinc, serum retinol, soluble transferrin receptor (sTfR), ferritin, and hemoglobin (Hb) status were collected at 7, 15, and 24 months of age of the children. For hemoglobin, one drop of capillary blood was collected and measured with the Hemocue device (Hb 201, Ängelholm, Sweden). Plasma zinc and ferritin levels were measured using Atomic Absorption Spectrometry and Chemiluminescence Immunoassay, respectively. sTfR levels were measured using immunoturbidimetry method. Hb, ferritin, and zinc values were adjusted for the presence of inflammation using alpha-1-acid glycoprotein values.^25^^,^^26^ Blood samples collected at 7 and 15 months of age was used for assessment of vaccine responses. Quantitative antimeasles, antitetanus toxoid, and antipertussis toxin IgG ELISAs (Euroimmun, Lubeck, Germany) and antipoliovirus IgG ELISAs (Genway) were performed according to the instructions of the manufacturer.^27^ During administering quantitative antirotavirus serum IgG and IgA ELISAs, microplates were coated with IgG antirotavirus rabbit, and either cell lysate or virus preparedness was applied to alternating rows after washing. Eight 2-fold dilutions were made, beginning with 1:80 dilutions of the IgA and IgG serum levels. Four 2-fold dilutions were prepared of 1:20 dilutions of known reference IgA and IgG and unknown serum or plasma samples. After washing, the microplates were inserted with the serum standard dilutions and serum sample dilutions. After washing again, biotinylated rabbit antihuman IgA (for the IgA plates) or IgG (for the IgG plates) was added and then avidin–biotin-peroxidase complex was washed and inserted. O-phenylenediamine dihydrochloride substrate was applied to each well after the final wash, and the reaction with sulfuric acid stopped. The plates were read at 492 nm, and a four-parameter fit of the transformed optical density values computed the titers.^28^

### Statistical analysis.

We first tabulated variables related to the household, maternal health, and child nutritional status using descriptive statistics (mean, standard deviation, and percentages, as appropriate). Then the group-wise distributions of biomarkers of enteric inflammation and vaccine titers were compared using box plots according to the outcome variables (PD [yes/no] and ND [yes/no]). Normality of the distribution of specific variables was determined using Shapiro-Wilk test. Student’s *t* test (for normally distributed variables) and Mann-Whitney *U* tests (for variables with skewed distribution) were applied to test the null hypothesis of no difference between the groups. Finally, multivariable logistic regression models were fit to identify the predictors of growth deviance. The binary outcome variables for the regression analyses were PD (yes/no) and ND (yes/no). Child-specific mean values of the predictor variables were used for fitting the regression models. The data sets that we used contain unequal clusters (Supplemental File 1; Table 2) of nonindependent observational units—namely, country. Statistically, measurements within a country might be more clustered than the measurements between the countries. To adjust this clustering effect, we used generalized linear mixed-effects models (GLMMs) where the intercept of the variable (i.e., country) was kept random. This approach allowed us to calculate more robust estimates of the variance in the outcome variable, both within and between the clusters.^29^ We built specific bivariate and multivariable logistic regression models for each of the outcome variables where GLMMs estimated the probability of being positive and negative deviants when a child is exposed to the predictor variables detailed above. The inclusion of children in different groups were done according to the definitions of PN, NPD, ND, and NND stated earlier. Initially, we conducted bivariate regression analyses (termed as unadjusted), and the variables showing statistically significant association (*P* < 0.05) to the specific outcome variables were selected for fitting the final multivariable models (termed *adjusted*). We reported the results of four multivariable models in Tables 2 and 3 (Table 2: factors associated with the chances of being positive and negative deviance; Table 3: association of vaccine titers to positive and negative deviances). Details of the model building process and the model statistics with random effects can be found in Supplemental File 2.

**Table 3:** Associations between levels of vaccine titers and the chances of being positive and negative deviants

Vaccine titers	Outcome variable: Positive deviant (yes vs. no)			
	Unadjusted		Adjusted*	
	Coefficient (95% CI)	*P* value	Coefficient (95% CI)	*P* value
Measles	−0.076 (–0.232 to 0.080)	0.338	−0.102 (–0.284 to 0.080)	0.273
Tetanus	0.151 (0.017 to 0.285)	0.027	0.182 (0.025 to 0.338)	0.023
Pertusis	0.023 (–0.113 to 0.159)	0.739	−0.139 (–0.306 to 0.027)	0.101
Rota	−0.007 (–0.126 to 0.112)	0.911	0.033 (–0.105 to 0.171)	0.639
Polio	0.139 (0.012 to 0.265)	0.031	0.137 (0.000 to 0.273)	0.050

	Outcome variable: Negative deviant (yes vs. no)			
	Unadjusted		Adjusted*	
	Coefficient (95% CI)	*P* value	Coefficient (95% CI)	*P* value
Measles	−0.039 (–0.121 to 0.043)	0.347	−0.021 (–0.113 to 0.071)	0.661
Tetanus	−0.027 (–0.089 to 0.035)	0.401	−0.049 (–0.126 to 0.028)	0.214
Pertusis	−0.013 (–0.078 to 0.053)	0.703	0.012 (–0.068 to 0.092)	0.773
Rota	−0.078 (–0.133 to −0.024)	0.005	−0.053 (–0.112 to 0.006)	0.077
Polio	−0.006 (–0.070 to 0.058)	0.854	0.014 (–0.057 to 0.084)	0.705

CI = confidence interval.

* Multivariable models were adjusted for sex, myeloperoxidase, neopterin, α-1-antitrypsin, length-for-age Z-score at birth, and WAMI index (Water, sanitation, hygiene, Asset, Maternal education, and Income index).

We carried out the data analysis in R (version 3.5.1), and the lme4 package was used for fitting the generalized linear mixed-effects models.^30^ A *P* value < 0.05 was considered as the margin of statistical significance for all the analyses.

## RESULTS

**Figure 1.**
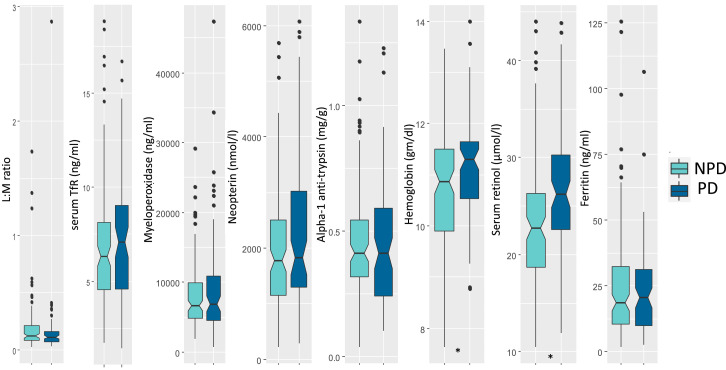
Box plots presenting distribution of biomarkers among the PD and NPD. Differences between the groups were tested with a Mann-Whitney test. L:M ratio in urine was higher in the NPD group. Concentrations of serum TfR, myeloperoxidase, neopterin, α-1 antitrypsin, and ferritin were all higher in the PD children, with the differences for hemoglobin and retinol levels achieving statistical significance (*P < 0.05). L:M ratio = lactulose-mannitol ratio; PD = positive deviant; NPD = nonpositive deviant; TfR = transferrin receptors. This figure appears in color at www.ajtmh.org.

**Figure 2.**
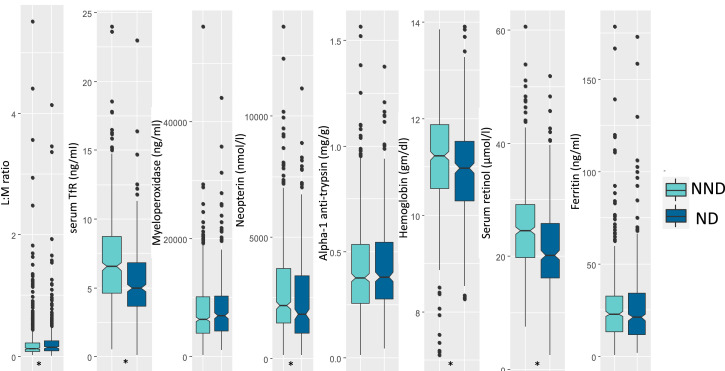
Box plots presenting distribution of biomarkers among the ND and NND. Differences between the groups were tested with a Mann-Whitney test. L:M ratio in urine was significantly (*P < 0.05) higher in the ND group. The concentrations of stool neopterine, serum TfR, hemoglobin, and retinol were lower in NDs achieving statistical significance (*P < 0.05). Concentrations of myeloperoxidase, and α-1 antitrypsin and ferritin were higher in the ND children. L:M ratio = lactulose-mannitol ratio; ND = negative deviant; NND = nonnegative deviant; TfR = transferrin receptors. This figure appears in color at www.ajtmh.org.

Table 1 presents the sociodemographic characteristics of the cohorts. Figures 1 and 2 present the levels of different biomarkers in positive and negative deviant groups. The median values for LM ratio (*P* value = 0.15) and ferritin (*P* value = 0.86) were lower in the positive deviant group while serum TfR (*P* value = 0.33), MPO (*P* value = 0.99), NEO (*P* value = 0.26), A1AT (*P* value = 0.26), Hb (*P* value = 0.011), and serum retinol (*P* value = 0.001) values were higher compared with their counterparts. Regarding the negative deviant group, the median values of LM (*P* value = 0.016), MPO (*P* value = 0.08), A1AT (*P* value = 0.47), and ferritin (*P* value = 0.39) were higher compared with their counterparts. On the other hand, serum TfR (*P* value < 0.001), NEO (*P* value < 0.001), Hb (*P* value < 0.001), and retinol (*P* value < 0.001) values were lower in negative deviants compared with the nonnegative deviant group.

**Figure 3.**
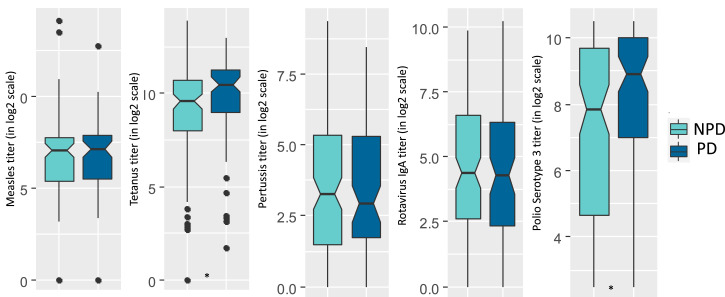
Box plots presenting distribution of different vaccine titers in PD and NPD groups. Differences between the groups were tested with a Mann-Whitney test. Titers of tetanus and polio serotype 3 were higher in the PD group achieving statistical significance (*P < 0.05). PD = positive deviant; NPD = nonpositive deviant. This figure appears in color at www.ajtmh.org.

**Figure 4.**
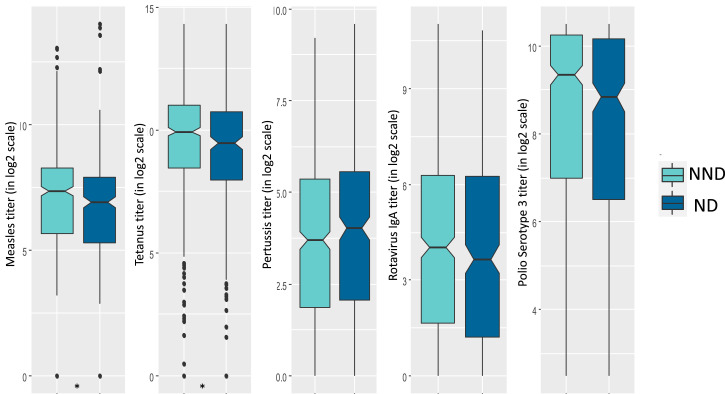
Box plots presenting distribution of different vaccine titers in ND and NND. Differences between the groups were tested with a Mann-Whitney test. Titers of measles and tetanus vaccines were lower in the negative deviant group achieving statistical significance (*P < 0.05). ND = negative deviant; NND = nonnegative deviant. This figure appears in color at www.ajtmh.org.

Figures 3 and 4 present the levels of different vaccine titers in positive and negative deviant groups. The median value for the titers of tetanus (*P* value = 0.012) was significantly higher in the positive deviants compared with their counterparts. On the other hand, the levels of measles (*P* value = 0.001) and tetanus (*P* value = 0.02) vaccine titers were significantly lower in the negative deviants than their nonnegative peers.

### Factors associated with the chances of being positive and negative deviants.

Table 2 shows the relationship between the factors associated with the chances of being positive and negative deviants in children. After fitting the multivariable logistic regression model, we found that WAMI score (regression coefficient, β = 1.54; 95% confidence interval [CI] = 0.80–2.28; *P* < 0.001), Hb (β = 0.36; 95% CI = 0.26–0.45; *P* < 0.001), retinol (β = 0.05; 95% CI = 0.04–0.07; *P* < 0.001), and LAZ score at birth (β = 0.47; 95% CI = 0.39–0.56; *P* < 0.001) were positively associated with positive deviance. Additionally, SRQ20 (β = –0.05; 95% CI = –0.07 to –0.02; *P* < 0.001), ferritin (β = –0.03; 95% CI = –0.03 to –0.02; *P* < 0.001), male sex (β = –1.08; 95% CI = –1.26 to –0.90; *P* < 0.001), A1AT (β = –0.81; 95% CI = –1.22 to –0.41; *P* < 0.001) were found to be negatively associated with the outcome. On the other hand, diarrhea episodes (β = 0.02; 95% CI = 0.01–0.03; *P* < 0.001), ferritin (β = 0.01; 95% CI = 0.01–0.01; *P* < 0.001), male sex (β = 0.72; 95% CI = 0.63–0.80; *P* < 0.001), and A1AT (β = 0.67; 95% CI = 0.48–0.86; *P* < 0.001) were positively associated, and energy from protein (β = –0.02; 95% CI = –0.03 to 0.00; *P* = 0.01), Hb (β = –0.28; 95% CI = –0.32 to –0.23; *P* < 0.001), TfR (β = –0.15; 95% CI = –0.17 to –0.13; *P* < 0.001), and LAZ score at birth (β = –0.90; 95% CI = –0.95 to –0.85; *P* < 0.001) were negatively associated with negative deviance after adjustment for the confounders. However, for both positive and negative deviants, levels of MPO and Neo were found to be associated to the outcome with a low coefficient value. The remaining factors did not produce any statistically significant (*P* > 0.05) results once the covariates were adjusted.

### Association between levels of vaccine titers and the chances of being positive and negative deviants.

Table 3 presents the association between different vaccine titers and the chances of becoming positive and negative deviants. The results of univariate logistic regression analyses found that tetanus (β = 0.151; 95% CI: 0.017–0.285; *P* = 0.027) and polio vaccine (β = 0.139; 95% CI = 0.012–0.265); *P* = 0.031) titers had statistically significant positive association to positive deviance and children with lower rotavirus vaccine titer had statistically significant higher chances (β = –0.078; 95% CI = –0.133 to –0.024; *P* = 0.005) of becoming negative deviant. The results of multivariable logistic regression analyses showed that tetanus vaccine titer was significantly positively associated with the chances of becoming positive deviant (β = 0.182; 95% CI = 0.025–0.338; *P* = 0.03) after adjusting for sex, WAMI score, MPO, Neo, A1AT and length-for-age Z-score at birth. No statistically significant association was seen between the rest of the vaccine titers with the outcome variables.

## DISCUSSION

We found that WAMI score, serum retinol, hemoglobin, LAZ score at birth, and tetanus and polio vaccine titers were positively and maternal depressive symptom score, serum ferritin, male sex, and α1-antitrypsin level were negatively associated with the likelihood of having a positively deviant linear growth status at 24 months of age. On the other hand, diarrhea episodes, male sex, α1-antitrypsin level were positively and hemoglobin, soluble transferrin receptor level, and LAZ score at birth were negatively associated to the chances of being negatively deviant.

It is well known that maternal depression interferes with mothers’ sense of responsibility and childrearing practices.^31^^,^^32^ Mothers’ better mental status could help a child regain the desired nutritional status at a later age, even if a child had a poor nutritional status at birth.^33^ The findings of our study echo this as we have found that maternal depressive symptom score had a statistically significant negative association with the likelihood of a child being a positive deviant. Several reports also showed the association between the commonly observed maternal mental disorders with childhood undernutrition in Bangladesh, Vietnam, and Ethiopia.^34^^,^^35^ Stewart et al. reported that sub‐Saharan African infants whose mothers were suffering from mental disorders were more likely to be stunted than underweight.^36^

In this study, we found that alpha-1-antitrypsin (A1AT) level was negatively associated with the chance of being positive deviant and positively associated with the probability of being negative deviant. A1AT is a protease inhibitor that protects cells from inflammatory proteases and clearance of A1AT is an useful marker of intestinal permeability and protein-losing enteropathy in children.^37^^,^^38^ Higher levels of A1AT in stool were found to be associated with lower catch-up growth and the levels also predicted declines in height-for-age Z-score for 6 months after the assessment.^39^^,^^40^ These elevations were reported to be associated with shigellosis and environmental enteropathy, two enteric conditions with the most pronounced impact on linear growth.^41^^,^^42^ Thus, the association of linear growth deviances with levels of A1AT supports the hypothesis that protein-losing enteropathy, an outcome of altered intestinal permeability is a prominent moderator of growth in infancy.

The association of hemoglobin, serum ferritin, and soluble transferrin receptor (sTfR) levels to the probability of becoming positive and negative deviants should be discussed in context of iron deficiency and nutrition-infection interaction. During the early months of life, a newborn rapidly uses its iron stores to support the accelerated growth and a corresponding increase in blood volume. The iron that a child receives from its mother is usually adequate for the first 4 to 6 months; afterward the iron demand rises rapidly with time to maintain sustained growth until the end of the second year of life and even beyond.^43^ During this stage of growth and development, if a child becomes iron deficient, his or her physical growth is impaired.^43^ Furthermore, even though the initial stage of iron depletion is asymptomatic, continued iron store depletion causes the storage level to become substantially reduced, and the hemoglobin synthesis begins to be affected, leading to iron deficiency. In the later stage, when iron stores are insufficient to maintain hemoglobin production, iron deficiency anemia ensues. This is why hemoglobin is a well-known tool for screening anemia status as a proxy to iron deficiency.^44^ Lower hemoglobin levels and iron deficiency decrease the oxygen-carrying capacity, thus compromising the energy production.^45^^,^^46^ As a result, the child becomes malnourished. This biological process supports our findings. In our analysis, we found that children with higher mean hemoglobin levels had a higher chance of becoming positive deviants, whereas children with lower hemoglobin levels had a higher tendency of becoming negative deviants. Our analysis also revealed that the chance of becoming negative deviant reduced with increased concentration of serum TfR levels. The transferrin receptor plays a critical role in iron metabolism by precisely controlling the flow of transferrin iron into body cells and reflects the total body mass of tissue receptors, which rise significantly in serum with tissue iron deficiency.^47^^,^^48^

In several published reports, serum ferritin, a quantitative measure of iron storage, was found to be increased in undernourished children.^49^^,^^50^ Plasma ferritin could be increased in a malnourished child who is suffering from frequent episodes of infection due to the disordered liver function.^51^^,^^52^ When such a child regains health, serum ferritin level might go back toward the physiological level. Similarly, we also found that children with lower serum levels of ferritin had a lower chance of becoming negative deviants and the direction of that relationship was opposite in the positive deviant group.

Recurrent infection impairs the nutrient balance of growing children and contributes to the pathogenesis of malnutrition.^4^ Vaccination, as an intervention in early life, can yield greater benefit in promoting healthy growth.^53^ Hence, the role of vaccination that might be critical in preventing malnutrition and promoting growth is worth exploring. We found a statistically significant positive association between tetanus and polio vaccine titers and chances of becoming positive deviant at the age of 24 months. Although several studies previously tried to describe malnutrition in the context of vaccination, to our knowledge, none of the studies evaluated the role of vaccination in linear growth deviances. Nevertheless, we found a small body of literature supporting the general hypothesis behind our findings. Anekwe et al. found that India’s universal immunization program reduced the height-for-age deficit among children under age 4 by 22% to 25% and the weight-for-age deficit by 15%.^54^

To the best of our knowledge, this is the first study of its kind to determine the factors responsible for positive and negative deviances in linear growth in children using multicountry birth cohort study data. The most novel aspect of this analysis is the differences and overlap between children who did not have perinatal/maternal risk factors but ended up being stunted and those even with the presence of the risk factors were able to recover from linear growth faltering. Moreover, we believe that the study design was rigorous, the data were robust, obtained by skilled field staff, and the laboratory analyses were done by expert personnel in sophisticated laboratories, which further fortify our findings.

However, we have several limitations to report. The etiology of linear growth faltering is widely recognized as an interaction between multiple physiological processes and any exploration of such phenomenon should be framed accordingly based on mucosal immune responses, history of maternal infection, and gestational age of the children. However, we could not explore the role of those variables due to unavailability of data. Our analysis also lacks information on intestinal pathogens, or gut microbiota, which are known to have potential roles in the underlying mechanism of gut enteropathy, as well as nutrient malabsorption. Lastly, the association between vaccine response and growth might not be a direct effect, but rather a confounding effect, and we might have failed to establish the causal relation due to lack of additional data.

In conclusion, our results suggested that enteric protein loss, micronutrient deficiency, vaccine responses, and maternal depressive symptoms were associated with linear growth deviances during the early years of childhood. Although children living in resource-limited settings frequently suffer from enteropathy and enteric protein loss, evidence is lacking on how to ameliorate such conditions to sustain optimal linear growth in children. Against such a background, we need a robust and effective public health approach to reduce the risk of intestinal inflammation and altered gut permeability in children. We also emphasize that, in addition to focusing on nutritional and biological factors, maternal mental health should also be considered carefully, especially for promoting better nutritional status in children in context of growth deviance.

## Supplemental Material


Supplemental materials
