# Supplementary material for: Associations of Enteric Protein Loss, Vaccine Response, Micronutrient Deficiency, and Maternal Depressive Symptoms with Deviance in Childhood Linear Growth: Results from a Multicountry Birth Cohort Study
Source: Am J Trop Med Hyg. 2022 Apr 11;106(6):1732–40. doi: 10.4269/ajtmh.21-0403 (PMC9209933; doi:10.4269/ajtmh.21-0403)
Supplement: Supplementary file 1 [file tpmd210403.SD1.pdf]

Supplemental Table 1:

Birth weight		Mother's height	Nutritional status at 24 months	Deviance type	n
<2.5 kg	and/or	<145 cm	Non-stunted	PD	79
<2.5 kg	and/or	<145 cm	Stunted	NPD	116
≥2.5 kg	and	≥145 cm	Stunted	ND	349
≥2.5 kg	and	≥145 cm	Non-stunted	NND	548
				Total	1092

Supplemental Table 2:

	PD		ND	
	No	Yes	No	Yes
Bangladesh	40	23	73	46
Brazil	0	12	130	6
India	34	26	95	68
Peru	25	14	100	52
South Africa	5	4	100	62
Tanzania	12	0	50	115
Total	116	79	548	349

Factors associated with the chances of being positive and negative deviance

Positive Deviance

Variables	Unadjusted					Variables	Adjusted				
	Beta	Std. error	95% CI		p value		Beta	Std. error	95% CI		p value
			UL	LL					UL	LL	
Energy from protein	0.03	0.00	0.03	0.02	0.000	Energy from protein	0.02	0.01	0.04	-0.01	0.186
Diarrhea episodes	-0.11	0.07	0.03	-0.26	0.118	Diarrhea episodes					
EBF Days	0.00	0.00	0.00	0.00	0.343	EBF Days					
WAMI	2.49	0.30	3.08	1.90	0.000	WAMI	1.54	0.38	2.28	0.80	0.000
SRQ20	-0.04	0.01	-0.02	-0.07	0.000	SRQ20	-0.05	0.01	-0.02	-0.07	0.000
LM Ratio	-0.48	0.15	-0.19	-0.76	0.001	LM Ratio	-0.14	0.18	0.21	-0.49	0.422
Hb	0.28	0.04	0.35	0.21	0.000	Hb	0.36	0.05	0.45	0.26	0.000
Zinc	0.02	0.02	0.06	-0.02	0.267	Zinc					
Retinol	0.07	0.01	0.08	0.06	0.000	Retinol	0.05	0.01	0.07	0.04	0.000
TFR	-0.01	0.01	0.02	-0.03	0.594	TFR					
Ferritin	-0.01	0.00	0.00	-0.01	0.001	Ferritin	-0.03	0.00	-0.02	-0.03	0.000
Sex	-0.90	0.08	-0.75	-1.05	0.000	Sex	-1.08	0.09	-0.90	-1.26	0.000
MPO	0.00	0.00	0.00	0.00	0.022	MPO	0.00	0.00	0.00	0.00	0.000
Neo	0.00	0.00	0.00	0.00	0.000	Neo	0.00	0.00	0.00	0.00	0.600
A1AT	-0.53	0.17	-0.19	-0.86	0.002	A1AT	-0.81	0.21	-0.41	-1.22	0.000
LAZ score at birth	0.33	0.04	0.40	0.26	0.000	LAZ score at birth	0.47	0.04	0.56	0.39	0.000

Random effects:

Groups Name Variance Std.Dev. Corr
country (Intercept) 1.125e+01 3.35399
month 2.676e-04 0.01636 1.00
Number of obs: 3039, groups: country, 6

Negative Deviance

Variables	Unadjusted					Variables	Adjusted				
	Beta	Std. error	95% CI		p value		Beta	Std. error	95% CI		p value
			UL	LL					UL	LL	
Energy from protein	-0.02	0.01	-0.01	-0.03	0.000	Energy from protein	-0.02	0.01	0.00	-0.03	0.006
Diarrhea episodes	0.01	0.01	0.02	0.00	0.011	Diarrhea episodes	0.02	0.01	0.03	0.01	0.000
EBF Days	0.00	0.00	0.00	0.00	0.000	EBF Days	0.00	0.00	0.00	0.00	0.000
WAMI	0.00	0.00	0.00	0.00	1.000	WAMI					
SRQ20	0.00	0.01	0.01	-0.01	0.875	SRQ20					
LM Ratio	0.04	0.05	0.14	-0.06	0.400	LM Ratio					
Hb	-0.21	0.02	-0.17	-0.25	0.000	Hb	-0.28	0.02	-0.23	-0.32	0.000
Zinc	0.00	0.01	0.02	-0.02	0.080	Zinc					
Retinol	-0.02	0.00	-0.01	-0.02	0.000	Retinol	0.00	0.00	0.00	-0.01	0.193
TFR	-0.06	0.01	-0.04	-0.07	0.000	TFR	-0.15	0.01	-0.13	-0.17	0.000
Ferritin	0.01	0.00	0.01	0.00	0.000	Ferritin	0.01	0.00	0.01	0.01	0.000
Sex	0.65	0.04	0.73	0.58	0.000	Sex	0.72	0.04	0.80	0.63	0.000
MPO	0.00	0.00	0.00	0.00	0.000	MPO	0.00	0.00	0.00	0.00	0.000
Neo	0.00	0.00	0.00	0.00	0.008	Neo	0.00	0.00	0.00	0.00	0.000
A1AT	0.50	0.09	0.67	0.33	0.000	A1AT	0.67	0.10	0.86	0.48	0.000
LAZ score at birth	-0.81	0.02	-0.76	-0.85	0.000	LAZ score at birth	-0.90	0.03	-0.85	-0.95	0.000

Random effects:

Groups Name Variance Std.Dev. Corr
country (Intercept) 1.463e+00 1.209708
month 1.622e-05 0.004027 -1.00
Number of obs: 13702, groups: country, 6

Association between levels of vaccine titres and the chances of being positive and negative deviant

	Outcome variable: PD (Yes vs. No)									
	Unadjusted					Adjusted*				
	B	SE	95% CI		p-value	B	SE	95% CI		p-value
			UL	LL				UL	LL	
Measles	-0.076	0.080	0.080	-0.232	0.338	-0.102	0.093	0.080	-0.284	0.273
Tetanus	0.151	0.068	0.285	0.017	0.027	0.182	0.080	0.338	0.025	0.023
pertusis	0.023	0.070	0.159	-0.113	0.739	-0.139	0.085	0.027	-0.306	0.101
Rota	-0.007	0.061	0.112	-0.126	0.911	0.033	0.070	0.171	-0.105	0.639
Polio	0.139	0.064	0.265	0.012	0.031	0.137	0.070	0.273	0.000	0.050

Random effects:

Groups Name Variance Std.Dev.
country (Intercept) 1.123855 1.06012
month 0.001951 0.04417
Number of obs: 193, groups: country, 6

	Outcome variable: ND (Yes vs. No)									
	Unadjusted					Adjusted*				
	B	SE	95% CI		p-value	B	SE	95% CI		p-value
			UL	LL				UL	LL	
Measles	-0.039	0.042	0.043	-0.121	0.347	-0.021	0.047	0.071	-0.113	0.661
Tetanus	-0.027	0.032	0.035	-0.089	0.401	-0.049	0.039	0.028	-0.126	0.214
pertusis	-0.013	0.033	0.053	-0.078	0.703	0.012	0.041	0.092	-0.068	0.773
Rota	-0.078	0.028	-0.024	-0.133	0.005	-0.053	0.030	0.006	-0.112	0.077
Polio	-0.006	0.032	0.058	-0.070	0.854	0.014	0.036	0.084	-0.057	0.705

Random effects:

Groups Name Variance Std.Dev. Corr
country (Intercept) 0.662771 0.81411
month 0.004892 0.06995 -1.00
Number of obs: 867, groups: country, 6

* Multi-variable models were adjusted for sex, WAMI score, MPO, Neo, A1AT and length-for-age Z-score at birth
